# Supplementary material for: Circular RNAs in Clear Cell Renal Cell Carcinoma: Their Microarray-Based Identification, Analytical Validation, and Potential Use in a Clinico-Genomic Model to Improve Prognostic Accuracy
Source: Cancers (Basel). 2019 Sep 30;11(10):1473. doi: 10.3390/cancers11101473 (PMC6826865; doi:10.3390/cancers11101473)
Supplement: Supplementary file 1 [file cancers-11-01473-s001.zip › cancers-581516-supplementary-final/cancers-581516-supplementary-final.docx]

**Supplementary Materials:**

[Supplementary Information S1: Sample size and power calculations 3](#_Toc20434023)

[Supplementary Information S2: RT-qPCR methodology 4](#_Toc20434024)

[**General comments** 4](#_Toc20434025)

[**Supplemental Table** **S1**. MIQE checklist according to Bustin et al. 4](#_Toc20434026)

[**cDNA synthesis** 8](#_Toc20434027)

[**qPCR- measurements** 8](#_Toc20434028)

[**Supplemental** **Table S2.** List of primers 8](#_Toc20434029)

[Determination of circEGLN3 and linEGLN3 9](#_Toc20434030)

[Determination of circNOX4 and linNOX4 10](#_Toc20434031)

[Determination of circRHOBTB3 and linRHOBTB3 11](#_Toc20434032)

[Determination of the reference genes PPIA and TBP 11](#_Toc20434033)

[**Performance data of the RT-qPCR analyses** 12](#_Toc20434034)

[**Supplemental Figure S1.** Specificity of the circRNA RT-PCR products 12](#_Toc20434035)

[Melting curve analyses 12](#_Toc20434036)

[Agarose gel electrophoresis of RT-qPCR products 14](#_Toc20434037)

[**Supplemental** **Table S3.** Characteristics of the PCR standard curves 15](#_Toc20434038)

[Supplementary Information S3: circRNA validation methods 16](#_Toc20434039)

[**Specific primer design for circRNAs** 16](#_Toc20434040)

[**Supplemental Table** **S4**. List of backsplice junctions and primers 16](#_Toc20434041)

[**RNAse R treatment of isolated total RNA** 16](#_Toc20434042)

[**Random hexamer primers vs. oligo(dT) primers for cDNA synthesis** 17](#_Toc20434043)

[**PCR products obtained using divergent and convergent primers** 17](#_Toc20434044)

[**Sanger-Sequencing of circRNA backsplice junction** 17](#_Toc20434045)

[Supplementary Information S4: Associations between clinicopathological variables and circRNAs/linear transcripts 18](#_Toc20434046)

[**Supplemental Table S5.** Associations of circRNAs and linear transcripts with clinicopathological variables 18](#_Toc20434047)

[**Supplemental Table S6A.** Correlation between circRNAs and linear transcripts in adjacent normal ccRCC tissue **18**](#_Toc20434048)

[**Supplemental Table S6B.** Correlation between circRNAs and linear transcripts in malignant ccRCC tissue samples 19](#_Toc20434049)

[**Supplemental Table S6C.** Significantly different correlation coefficients of circRNAs and linear transcripts between malignant and adjacent normal samples 19](#_Toc20434050)

[Supplementary Information S5: Cox regressions and Kaplan-Meier curves 20](#_Toc20434051)

[**Supplemental Table S7**. Univariate Cox regressions of circRNAs and linRNAs 20](#_Toc20434052)

[**Supplemental Figure S2A–B**. Kaplan-Meier curves of circRNAs and linRNAs 21](#_Toc20434053)

[**Supplemental Figure S3A–C**. Kaplan-Meier analysis using TCGA data 22](#_Toc20434054)

[**Supplemental Table S8**. Univariate Cox regressions of clinicopathological factors 23](#_Toc20434055)

[**Supplemental Figure S4A–C**. Kaplan-Meier curves of clinicopathological factors 24](#_Toc20434056)

[**Supplemental Table S9**. Bootstrapping *p*-values of Cox regression analyses 27](#_Toc20434057)

[References 28](#_Toc20434058)

**Supplemental Microarray Excel File (separate file)**

Supplementary Information S1: Sample size and power calculations

The clinical decision points refer to the diagnostic and prognostic usefulness of the circRNAs in combination with their linear transcription counterparts as well as with the standard clinical variables. To evaluate the clinical validity of the markers with respect to avoid both type I and type II errors, the conventional thresholds of α=5% (significance level) and β=20% (1-power; power of 80%) were selected for sample size calculation. The calculations were performed using the statistics programs MedCalc 19.0.6 (MedCalc Software bvba, Ostend, Belgium), GPower 3.1.9.4 (Franz Faul; Kiel University, Kiel, Germany), and GraphPad StatMate 2.0 (GraphPad Software, San Diego, CA, USA).

The necessary sample size to test the discriminative ability of the markers to differentiate between matched pairs of malignant and adjacent normal tissue was performed on the basis of receiver-operating characteristics (ROC) analysis. A sufficient discrimination could be assumed with an area under the ROC curve (AUC) of 0.7. Under these conditions and taken into account the above-mentioned power criteria, a sample size of 31 pairs would necessary to be included in this study.

The clinical endpoints to assess the prognostic validity were cancer-specific survival, recurrence-free survival, and overall survival. As the main clinical endpoint in this study was the cancer-specific survival, we calculated the necessary sample size based on a difference of cancer-specific survival rates of 0.30 in the Kaplan-Meier curves for the examined variable/marker. At a ratio of three between patients with cancer-specific death and censored data, a total sample size of 80 would be required. According to these sample size calculations, we concluded that at least 90 tumor patients should be included in this study for evaluating especially the prognostic capacity of the new genomic markers of interest.

Therefore, the final inclusion of 99 malignant ccRCC tissue samples and 85 samples from adjacent normal tissue can be considered as a suitable sample size to provide statistically reliable results.

Supplementary Information S2: RT-qPCR methodology

RT-qPCR measurements were performed according to the recommendations of the MIQE guidelines [1]. No template controls (NTC) and no reverse transcription controls (NRTC or no enzyme controls=NEC) were always performed and showed negative results. The corresponding comments are listed in the following checklist (Supplemental Table S1) and apply for all assays. Quantitative PCR data analysis was done using qbase+ software, version 3.2 (Biogazelle, Zwijnaarde, Belgium; www.qbaseplus.com).

**Table S1.** MIQE checklist according to Bustin et al.

| **Item to Check** | **Importance** | **Checklist** | **Where; Comment** |
| --- | --- | --- | --- |
| **Experimental Design** | | | |
| Definition of experimental and control groups | **E** | Yes | Main text: Materials and Methods; Results: Table 1 |
| Number within each group | **E** | Yes | Main text: Materials and Methods; Results: Table 1 |
| Assay carried out by core lab or investigator's lab? | D | Yes | Investigator's lab |
| Acknowledgement of authors' contributions | D | No | Yes; section Acknowledgements |
| **Sample** | | | |
| Description | **E** | Yes | Main text: Results; Materials and Methods. |
| Volume/mass of sample processed | D | Yes | Main text: Results; Materials and Methods |
| Microdissection or macrodissection | **E** | Yes | Main text: Materials and Methods |
| Processing procedure | **E** | Yes | Main text: Materials and Methods |
| If frozen - how and how quickly? | **E** | Yes | Main text: Materials and Methods |
| If fixed - with what, how quickly? | **E** | Yes | Main text: Materials and Methods |
| Sample storage conditions and duration (esp. for FFPE samples) | **E** | Yes | Main text: Materials and Methods |
| **Nucleic Acid Extraction** | | | |
| Procedure and/or instrumentation | **E** | Yes | Main text: Materials and Methods |
| Name of kit and details of any modifications | **E** | Yes | Main text: Materials and Methods |
| Source of additional reagents used | D | Not applicable |  |
| Details of DNase or RNase treatment | **E** | Yes | Main text: Materials and Methods: RNA extraction, on-column DNase digestion |
| Contamination assessment (DNA or RNA) | **E** | Yes | Supplementary Information S2: General comments: genomic DNA contamination was excluded by control experiments without reverse transcription for all targets |
| Nucleic acid quantification | **E** | Yes | Main text: Materials and Methods |
| Instrument and method | **E** | Yes | Main text: Materials and Methods; Supplementary Information S2 |
| Purity (A260/A280) | D | Yes | Main text: Materials and Methods |
| Yield | D | Yes | Main text: Materials and Methods |
| RNA integrity method/instrument | **E** | Yes | Main text: Materials and Methods: RIN; Agilent |
| RIN/RQI or Cq of 3' and 5' transcripts | **E** | Yes | Main text: Materials and Methods: RIN; Agilent |
| Electrophoresis traces | D | No |  |
| Inhibition testing (Cq dilutions, spike or other) | **E** | Yes | Supplementary Information 2: Cq dilution, see standard curve characteristics in Supplemental Table S3 |
| **Reverse Transcription** | | | |
| Complete reaction conditions | **E** | Yes | Main text: Materials and Methods; Supplementary Information S2: RT-qPCR methodology, cDNA synthesis |
| Amount of RNA and reaction volume | **E** | Yes | Main text: Materials and Methods; Supplementary Information S2: RT-qPCR methodology, cDNA synthesis |
| Priming oligonucleotide (if using GSP) and concentration | **E** | Yes | Main text: Materials and Methods; Supplementary Information S2: RT-qPCR methodology, cDNA synthesis |
| Reverse transcriptase and concentration | **E** | Yes | Main text: Materials and Methods; Supplementary Information S2: RT-qPCR methodology, cDNA synthesis |
| Temperature and time | **E** | Yes | Main text: Materials and Methods; Supplementary Information S2: RT-qPCR methodology, cDNA synthesis |
| Manufacturer of reagents and catalogue numbers | D | Yes | Main text: Materials and Methods; Supplementary Information S2: RT-qPCR methodology, cDNA synthesis |
| Cqs with and without RT | D | Yes | Supplementary Information S2: RT-qPCR methodology: neg. results; see also comment on DNase treatment |
| Storage conditions of cDNA | D | Yes | Main text. Materials and Methods; storage at -80°C |
| **qPCR Target Information** | | | |
| Gene symbols | **E** | Yes | Main text: Results; Table 2 |
| If multiplex, efficiency and LOD of each assay. | **E** | Not applicable |  |
| Sequence accession number | **E** | Yes | Main text: Results, Table 2; Supplementary Information 3, Supplemental Table S4. Supplemental Micorarray Excel File |
| Location of amplicon | D | Yes | Supplementary Information 3, Supplemental Table S4. Supplemental Microarray Excel File |
| Amplicon length | **E** | Yes | Supplementary Information S2, RT-qPCR methodology with Supplemental Figure S1: Agarose electrophoresis. Main text: Results, Figure 3C |
| *In silico* specificity screen (BLAST, etc) | **E** | Yes | Main text: Materials and Methods; Supporting Information S2, RT-qPCR methodology |
| Pseudogenes, retropseudogenes or other homologs? | D | Yes | Supplementary Information S2: RT-qPCR methodology. Supplementary Information S3: Analysis of data base circBAse, Supplemental Table S4 |
| Sequence alignment | D | Yes | Supplementary Information S2: RT-qPCR methodology. Supplementary Information S3: Analysis of data base circBAse, Supplemental Table S4 |
| Secondary structure analysis of amplicon | D | No |  |
| Location of each primer by exon or intron (if applicable) | **E** | Yes | Supplementary Information S2: RT-qPCR methodology. Supplementary Information S3: Analysis of data base circBAse, Supplemental Table S4. Supplemental Microarray Excel File |
| What splice variants are targeted? | **E** | Yes | Supplementary Information S2: RT-qPCR methodology. Supplementary Information S3: Analysis of data base circBAse, Supplemental Table S4. Supplemental Excel Microarray Excel File |
| **qPCR Oligonucleotides** | | | |
| Primer sequences | **E** | Yes | Supplementary Information S2: RT-qPCR methodology with Supplemental Table S2. Supplementary Information S3, Supplemental Table S4 (specific primer design for circRNAs) |
| RTPrimerDB Identification Number | D | No |  |
| Probe sequences | D | Yes | Supplementary Information S2: RT-qPCR methodology, see only for determination of linear NOX4 |
| Location and identity of any modifications | **E** | Yes | Supplementary Information S2: RT-qPCR methodology |
| Manufacturer of oligonucleotides | D | Yes | TIB MolBiol (Berlin, Germany) |
| Purification method | D | Yes | TIB MolBiol: probes HPLC, primers HPLC or GSF purification; other unknown (PPIA: test kit) |
| **qPCR Protocol** | | | |
| Complete reaction conditions | **E** | Yes | Main text: Materials and Methods. Supplementary Information S2: RT-qPCR methodology |
| Reaction volume and amount of cDNA/DNA | **E** | Yes | Main text: Materials and Methods. Supplementary Information S2: RT-qPCR methodology |
| Primer, (probe), Mg++ and dNTP concentrations | **E** | Yes | Main text: Materials and Methods. Supplementary Information S2: RT-qPCR methodology |
| Polymerase identity and concentration | **E** | Yes | Main text: Materials and Methods. Supplementary Information S2: RT-qPCR methodology |
| Buffer/kit identity and manufacturer | **E** | Yes | Main text: Materials and Methods. Supplementary Information S2: RT-qPCR methodology |
| Exact chemical constitution of the buffer | D | No | The manufacturers does not provide this information |
| Additives (SYBR Green I, DMSO, etc.) | **E** | Yes | Main text: Materials and Methods. Supplementary Information S2: RT-qPCR methodology |
| Manufacturer of plates/tubes and catalogue number | D | Yes | Supplementary Information S2: RT-qPCR methodology (Roche; Cat.No. 04729692001) |
| Complete thermocycling parameters | **E** | Yes | Main text: Materials and Methods; Supplementary Information S2: RT-qPCR methodology |
| Reaction setup (manual/robotic) | D | Yes | Manual setup |
| Manufacturer of qPCR instrument | **E** | Yes | Main text: Materials and Methods: LightCycler 480 (Roche) |
| **qPCR Validation** | | | |
| Evidence of optimisation | D | Yes | Supplementary Information S2: RT-qPCR methodology with Supplemental Figure S1 (melting curves; agarose electrophoresis) |
| Specificity (gel, sequence, melt, or digest) | **E** | Yes | Main text: Results, Figure 3 with all circRNA validation data. Supplementary Information S2: RT-qPCR methodology with Supplemental Figure S1. Supplementary Information 3 with validation methods of circRNAs |
| For SYBR Green I, Cq of the NTC | **E** | Yes | Supplementary Information S2: RT-qPCR methodology, general comments |
| Standard curves with slope and y-intercept | **E** | Yes | Supplementary Information S2: RT-qPCR methodology with Supplemental Table S3 |
| PCR efficiency calculated from slope | **E** | Yes | Supplementary Information S2: RT-qPCR methodology with Supplemental Table S3 |
| Confidence interval for PCR efficiency or standard error | D | Yes | Supplementary Information S2: RT-qPCR methodology with Supplemental Table S3 |
| r2 of standard curve | **E** | No | Not provided by the LC480 software |
| Linear dynamic range | **E** | Yes | Supplementary Information S2: RT-qPCR methodology with Supplemental Table S3 |
| Cq variation at lower limit | **E** | Yes | Supplementary Information S2: RT-qPCR methodology, Supplemental Table S3 with Cq range of the measured samples |
| Confidence intervals throughout range | D | No |  |
| Evidence for limit of detection | **E** | Yes | Supplementary Information S2: RT-qPCR methodology, Supplemental Table S3: samples with Cq values in the dynamic range of the standard curves |
| If multiplex, efficiency and LOD of each assay. | **E** | Not applicable |  |
| **Data Analysis** | | | |
| qPCR analysis program (source, version) | **E** | Yes | Main text: Materials and Methods. Supplementary Information S2: RT-PCR methodology with Supplemental Table S3 (LightCycler software, release 1.5.0 using the “second derivative maximum” method); qbase+ software, version 3.2 (Biogazelle, Zwijnaarde, Belgium) |
| Cq method determination | **E** | Yes |  |
| Outlier identification and disposition | **E** | Not applicable |  |
| Results of NTCs | **E** | Yes | Supplementary Information S2: RT-PCR methodology, General comments |
| Justification of number and choice of reference genes | **E** | Yes | Main text: Materials and Methods; Results. Supplementary Information S1 and Supplementary Information 2: RT-PCR methodology with reference genes PPIA and TBP according to [2] |
| Description of normalisation method | **E** | Yes | Main text: Results, Legend to Figure 4. Supplementary Information S2: RT-PCR methodology: reference genes PPIA and TBP |
| Number and concordance of biological replicates | D | Yes | Main text: Table 1; Figure 4 |
| Number and stage (RT or qPCR) of technical replicates | **E** | Yes | Supplementary Information S2: RT-PCR methodology, at least technical duplicates |
| Repeatability (intra-assay variation) | **E** | Yes | Main text: Results, Table 3 |
| Reproducibility (inter-assay variation, %CV) | D | No | Main text: Results, Table 3 |
| Power analysis | D | Yes | Main text: Materials and Methods; Results. Supplementary Information S1 |
| Statistical methods for result significance | **E** | Yes | Main text: Materials and Methods: Statistics and Data Analysis. Results: Legends of Figures and Tables |
| Software (source, version) | **E** | Yes | Main text: Materials and Methods: Statistics and Data Analysis. Supplementary Information S1 |
| Cq or raw data submission using RDML | D | No |  |

In the following sections, the cDNA synthesis and quantification of circRNAs, linRNAs, and the reference genes PPIA and TBP including the analytical performance data for all the measurements are compiled.

cDNA synthesis

Maxima First Strand cDNA Synthesis Kit for RT-qPCR (Thermo Fisher Scientific, Waltham, MA, USA; Cat.No. K1642) was used in final reaction volume of 20 µL according to the following protocol:

| **Volume (µL)** | **Reagent/Sample** | **Components** |
| --- | --- | --- |
| 4 | 5X Reaction Mix | Reaction buffer, dNTPs, oligo(dT)18 and random hexamer primers |
| 2 | Maxima Enzyme Mix | Maxima Reverse Transcriptase (RT) and  Thermo Scientific™ RiboLock™ RNase Inhibitor |
| 2 | Total RNA (1 µg) | Diluted RNA (gDNA free); see RNA isolation |
| 12 | Water, nuclease free |  |

The transcription reaction was carried out in a thermal block cycler with heated lid (Biometra GmbH, Göttingen, Germany) as follows: 10 min at 25°C, followed by 5 min at 50°C and terminated by heating at 85°C for 5 min; end 4°C. All cDNA samples were stored at -80°C until qPCR analysis.

qPCR- measurements

All real-time qPCR runs were performed on the LightCycler 480 Instrument (Roche Molecular Diagnostics, Mannheim, Germany) in white 96-well plates (Cat.No. 04729692001) using at least technical duplicates and resulting mean values for further calculations. Maxima SYBR Green qPCR Master Mix (2X) (Thermo Fisher Scientific; Cat.No. K0252) was used. Primers are compiled in the following Supplemental Table S2 and were synthesized by TIB MOLBIOL GmbH (Berlin, Germany) unless otherwise specified. PPIA (peptidylprolyl isomerase A) and TBP (TATA-box binding protein) were used as normalizers [2]. Quantitative PCR data analysis was done using qbase+ software, version 3.2 (Biogazelle, Zwijnaarde, Belgium; www.qbaseplus.com).

**Table 2.** List of primers.

| **circRNA (circBase ID)** | **Primer name** | **Primer sequence** |
| --- | --- | --- |
| circEGLN3 (hsa_circ_0101692) | div_circEGLN3_forward | TCCTGCAGACATCCTACTCG |
| circEGLN3 (hsa_circ_0101692) | div_circEGLN3_reverse | GATGCAGCGACCATCACC |
| circNOX4 (hsa_circ_0023984) | div_circNOX4_forward | CATTGTCCCAGTGTATCTGCAT |
| circNOX4 (hsa_circ_0023984) | div_circNOX4_reverse | TGAGAGCTGGTTCGGTTAAGA |
| circRHOBTB3 (hsa_circ_0007444) | div_circRHOBTB3_forward | TTCTGGGGATGTTTCAAATG |
| circRHOBTB3 (hsa_circ_0007444) | div_circRHOBTB3_reverse | ACACACTGGCAGCAGAACAG |
| circEGLN3 (hsa_circ_0101692) | con_circEGLN3_forward | AGACTGACCGTGCTCTGAAA |
| circEGLN3 (hsa_circ_0101692 | con_circEGLN3_reverse | ACAGGGATGTGAAGGATGCA |
| circNOX4 (hsa_circ_0023984) | con_circNOX4_forward | AACCGAACCAGCTCTCAGAA |
| circNOX4 (hsa_circ_0023984) | con_circNOX4_reverse | AGCTTGGAATCTGGGCTCTT |
| circRHOBTB3 (hsa_circ_0007444) | con_circRHOBTB3_forward | AGATCGTTCTCTGCGCTGTA |
| circRHOBTB3 (hsa_circ_0007444) | con_circRHOBTB3_reverse | TCATGGCTAGCACCTGGAAA |
| circRNA4 ^a^ (hsa_circ_0001900) | hsa_circRNA4_forward | TGTGCTCCTGCTCATACTGGTCAA |
| circRNA4 ^a^ (hsa_circ_0001900) | hsa_circRNA4_reverse | TCAGTGCCTCGAAAGAACTTCCGT |
| circRNA9 ^a^ (hsa_circ_0001423) | hsa_circRNA9_forward | GCTCTCCAAAAAGGGGAATC |
| circRNA9 ^a^ (hsa_circ_0001423) | hsa_circRNA9_reverse | CCCCTGAACTGAAACCACTG |
| **Linear transcript (NCBI Genbank)** |  | |
| NM_022073.3 | hsa_linEGLN3_forward | CTGTCTGGTACTTTGATGCTGAA |
| NM_022073.3 | hsa_linEGLN3_reverse | TCAGTGAGGGCAGATTCAGTT |
| NM_016931.4 ^b^ | hsa_linNOX4_forward | AACCAAGGGCCAGAGTATCA |
| NM_016931.4 ^b^ | hsa_linNOX4_reverse | GCTGAGGCTCTGCTTAGACAC |
| NM_014899.3 | hsa_linRHOBTB3_forward | CCACCTCAACTTGAACAACCA |
| NM_014899.3 | hsa_linRHOBTB3_reverse | GGCAGCAGAACAGCAAGTTA |
| NM_002046.5 | hsa_GAPDH_forward | AAGGTGAAGGTCGGAGTCAAC |
| NM_002046.5 | hsa_GAPDH_reverse | GGGGTCATTGATGGCAACAATA |
| PPIA^c^ | hsa_PPIA_forward | QuantiTect Primer Assay (QT00052311) |
| PPIA^c^ | hsa_PPIA_reverse | QuantiTect Primer Assay (QT00052311) |
| NM_003194.4 | hsa_TBP_forward | TTCGGAGAGTTCTGGGATTGTA |
| NM_003194.4 | hsa_TBP_reverse | TGGACTGTTCTTCACTCTTGGC |
| NM_003373.3 | hsa_VCL_forward | CTCGTCCGGGTTGGAAAAGAG |
| NM_003373.3 | hsa_VCL_reverse | AGTAAGGGTCTGACTGAAGCAT |
| NM_001363876.1 | hsa_TMEM_forward | GCGGTCAAGTCTCATTCTGC |
| NM_001363876.1 | hsa_TMEM_reverse | GGGGATACAGGACAAATCCAA |

^a^ According to Memczak et *al.* [3]. ^b^ With UPL Probe #22. ^c^ Detection of NM_001008741;NM_021130;NM_203430;NM_203431;NM_001300981.

Determination of circEGLN3 and linEGLN3

Reaction setup (for circEGLN3 and linEGLN3).

| **Volume (µL)** | **Reagent/Sample** | **Components** |
| --- | --- | --- |
| 5 | Maxima SYBR Green qPCR Master Mix (2X) | Maxima Hot Start Taq DNA Polymerase, dNTPs (also dUTP) and SYBR Green I in an optimized PCR buffer |
| 2 | Primer Mix | Forward and reverse primer mix, final concentration 0.250 µM |
| 1 | cDNA | Prediluted (1:10) |
| 2 | Water, nuclease free |  |
| **Total volume 10 µL** | |  |

LightCycler 480 measurement conditions circEGLN3.

| **Setup** | | | | | |
| --- | --- | --- | --- | --- | --- |
| **Block type** | | | Reaction volume (µL) | | |
| **96** | | | 10 | | |
| **Detection format** | | Excitation filter |  | Emission filter | |
| SYBR Green | | 465 nm | | 510 nm | |
| **Programs** | | | | | |
| Program names | | Cycles | | Analysis mode | |
| Pre-incubation | | 1 | | None | |
| Amplification | | 45 | | Quantification | |
| Melting curve | | 1 | | Melting curve | |
| Cooling | | 1 | | None | |
| **Temperature targets** | | | | | |
|  | Target (°C) | Acquisition mode | Hold time (s) | Ramp rate (°C/s) | Acquisitions (per °C) |
| Pre-incubation | 95 | None | 600 | 4.4 | - |
| Amplification | 95 | None | 15 | 4.4 | - |
|  | 60 | None | 30 | 2.2 | - |
|  | 79 | Single | 2 | 4.4 | - |
| Melting curve | 95 | None | 5 | 4.4 | - |
|  | 65 | None | 60 | 2.2 | - |
|  | 95 | Continuous | - | 0.11 | 5 |
| Cooling | 40 | None | 30 | 1.5 | - |

linEGLN3 (conditions corresponding to circRNA except for temperature targets).

| **Temperature targets** | | | | | |
| --- | --- | --- | --- | --- | --- |
|  | **Target (°C)** | **Acquisition mode** | **Hold time (s)** | **Ramp rate (°C/s)** | Acquisitions (per °C) |
| Pre-incubation | 95 | None | 600 | 4.4 | - |
| Amplification | 95 | None | 15 | 4.4 | - |
|  | 60 | None | 30 | 2,2 | - |
|  | 70 | Single | 15 | 4.4 | - |
| Melting curve | 95 | None | 5 | 4.4 | - |
|  | 65 | None | 60 | 2.2 | - |
|  | 95 | Continuous | - | 0.11 | 5 |
| Cooling | 40 | None | 30 | 1.5 | - |

Determination of circNOX4 and linNOX4

Reaction setup for circNOX4 according to the protocol of circEGLN3 (with specific primers).

LightCycler 480 measurement conditions.

| **Setup** | | | | | |
| --- | --- | --- | --- | --- | --- |
| **Block type** | | | Reaction Volume (µL) | | |
| **96** | | | 10 | | |
| **Detection format** | | Excitation filter |  | Emission filter | |
| SYBR Green | | 465 nm | | 510 nm | |
| **Programs** | | | | | |
| Program names | | Cycles | | Analysis mode | |
| Pre-incubation | | 1 | | None | |
| Amplification | | 45 | | Quantification | |
| Melting curve | | 1 | | Melting curve | |
| Cooling | | 1 | | None | |
| **Temperature targets** | | | | | |
|  | Target (°C) | Acquisition mode | Hold time (s) | Ramp rate (°C/s) | Acquisitions (per °C) |
| Pre-incubation | 95 | None | 600 | 4.4 | - |
| Amplification | 95 | None | 15 | 4.4 | - |
|  | 60 | None | 15 | 1.0 | - |
|  | 70 | Single | 15 | 4.4 | - |
| Melting curve | 95 | None | 5 | 4.4 | - |
|  | 65 | None | 60 | 2.2 | - |
|  | 97 | Continuous | - | 0.11 | 5 |
| Cooling | 40 | None | 30 | 1.5 | - |

linNOX4.

Reaction setup (LightCycler 480 Probes Master (Roche, Cat.No. 04707494001).

| **Volume (µL)** | **Reagent/Sample** | **Components** |
| --- | --- | --- |
| 5 | LightCycler Probes Master |  |
| 1 | Primer Mix | Forward and reverse primer mix, final concentration 0.125 µM |
| 1 | UPL Probe #22 | Prediluted, protocol Roche |
| 1 | cDNA | Prediluted (1:10) |
| 2 | Water, nuclease free |  |
| **Total volume 10 µL** | |  |

LightCycler 480 measurement conditions.

| **Setup** | | | | | |
| --- | --- | --- | --- | --- | --- |
| **Block type** | | | Reaction Volume (µL) | | |
| **96** | | | 10 | | |
| **Detection format** | | Excitation filter |  | Emission filter | |
| Mono Color Hydrolysis Probe | | 465 nm | | 510 nm | |
| **Programs** | | | | | |
| Program names | | Cycles | | Analysis mode | |
| Pre-incubation | | 1 | | None | |
| Amplification | | 45 | | Quantification | |
| Cooling | | 1 | | None | |
| **Temperature targets** | | | | | |
|  | Target (°C) | Acquisition mode | Hold time (s) | Ramp rate (°C/s) | Acquisitions (per °C) |
| Pre-incubation | 95 | None | 600 | 4.4 |  |
| Amplification | 95 | None | 10 | 4.4 |  |
|  | 60 | None | 30 | 2.2 |  |
|  | 72 | Single | 1 | 4.4 |  |
| Cooling | 40 | None | 60 | 1.5 |  |

Determination of circRHOBTB3 and linRHOBTB3

**Reaction setup** for both RHOBTB3s according to the protocol of circEGLN3 (with specific primers).

LightCycler measurement conditions for both RHOBTB3s.

| **Setup** | | | | | |
| --- | --- | --- | --- | --- | --- |
| **Block type** | | | Reaction Volume (µL) | | |
| **96** | | | 10 | | |
| **Detection format** | | Excitation filter |  | Emission filter | |
| SYBR Green | | 465 nm | | 510 nm | |
| **Programs** | | | | | |
| Program names | | Cycles | | Analysis mode | |
| Pre-incubation | | 1 | | None | |
| Amplification | | 45 | | Quantification | |
| Melting curve | | 1 | | Melting curve | |
| Cooling | | 1 | | None | |
| **Temperature targets** | | | | | |
|  | Target (°C) | Acquisition mode | Hold time (s) | Ramp rate (°C/s) | Acquisitions (per °C) |
| Pre-incubation | 95 | None | 600 | 4.4 | - |
| Amplification | 95 | None | 15 | 4.4 | - |
|  | 60 | None | 15 | 1.0 | - |
|  | 70 | Single | 15 | 4.4 | - |
| Melting curve | 95 | None | 5 | 4.4 | - |
|  | 65 | None | 60 | 2.2 | - |
|  | 95 | Continuous | - | 0.11 | 5 |
| Cooling | 40 | None | 30 | 1.5 | - |

Determination of the reference genes PPIA and TBP

Reaction setup for PPIA.

| **Volume (µL)** | **Reagent/Sample** | **Components** |
| --- | --- | --- |
| 5 | Maxima SYBR Green qPCR Master Mix (2X) | Maxima Hot Start Taq DNA Polymerase, dNTPs (also dUTP) and SYBR Green I in an optimized PCR buffer |
| 2 | Primer Mix | Forward and reverse primer mix (1x) according to the protocol of the Hs PPIA_1_SG QuantiTect Primer Assay (Qiagen; Cat. No. QT00052311) |
| 1 | cDNA | Prediluted (1:10) |
| 2 | Water, nuclease free |  |
| **Total volume 10 µL** | |  |

Reaction setup for TBP.

| **Volume (µL)** | **Reagent/Sample** | **Components** |
| --- | --- | --- |
| 5 | Maxima SYBR Green qPCR Master Mix (2X) | Maxima Hot Start Taq DNA Polymerase, dNTPs (also dUTP) and SYBR Green I in an optimized PCR buffer |
| 2 | Primer Mix | Forward and reverse primer mix, final concentration 0.250 µM |
| 1 | cDNA | Prediluted (1:10) |
| 2 | Water, nuclease free |  |
| **Total volume 10 µL** | |  |

LightCycler measurement conditions for PPIA and TBP.

| **Setup** | | | | | |
| --- | --- | --- | --- | --- | --- |
| **Block type** | | | Reaction Volume (µL) | | |
| **96** | | | 10 | | |
| **Detection format** | | Excitation filter |  | Emission filter | |
| SYBR Green | | 465 nm | | 510 nm | |
| **Programs** | | | | | |
| Program names | | Cycles | | Analysis mode | |
| Pre-incubation | | 1 | | None | |
| Amplification | | 45 | | Quantification | |
| Melting curve | | 1 | | Melting curve | |
| Cooling | | 1 | | None | |
| **Temperature targets** | | | | | |
|  | Target (°C) | Acquisition mode | Hold time (s) | Ramp rate (°C/s) | Acquisitions (per °C) |
| Pre-incubation | 95 | None | 900 | 4.4 | - |
| Amplification | 95 | None | 15 | 4.4 | - |
|  | 58 | None | 20 | 1.0 | - |
|  | 72 | None | 20 | 4.4 |  |
|  | 79 | Single | 2 | 4.4 | - |
| Melting curve | 92 | None | 5 | 4.4 | - |
|  | 65 | None | 60 | 2.2 | - |
|  | 95 | Continuous | - | 0.11 | 5 |
| Cooling | 40 | None | 60 | 1.5 | - |

Performance data of the RT-qPCR analyses

A)


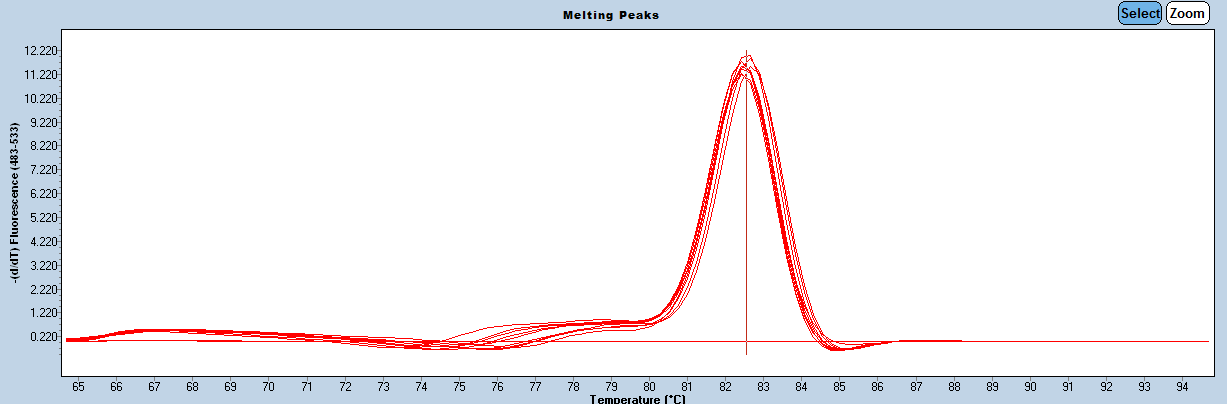


B)


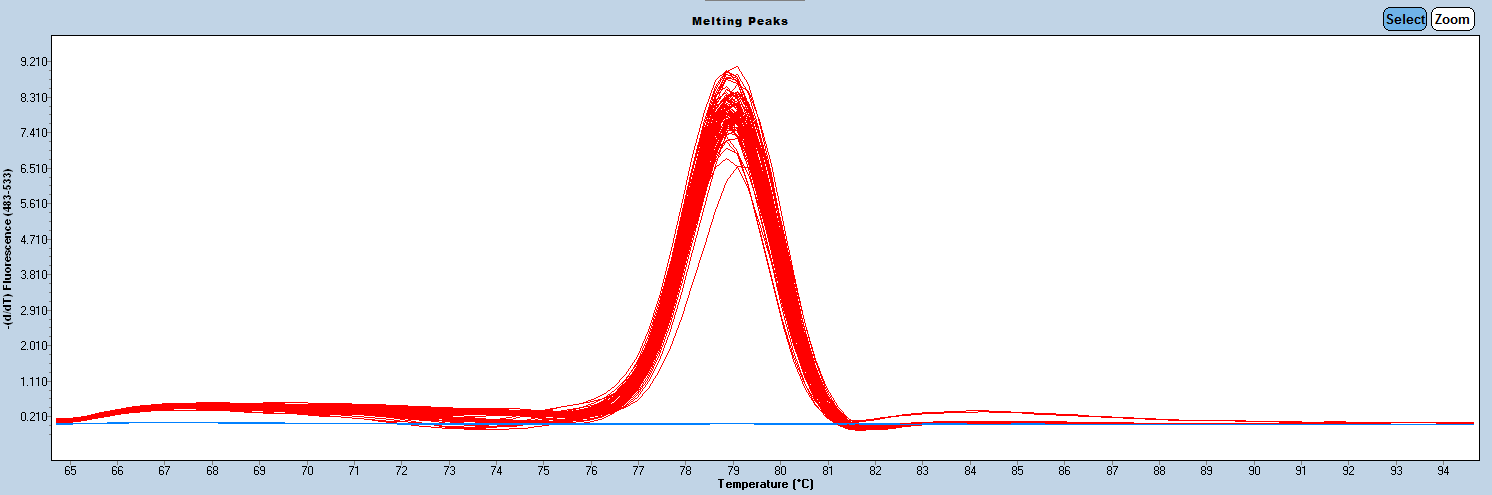


C)


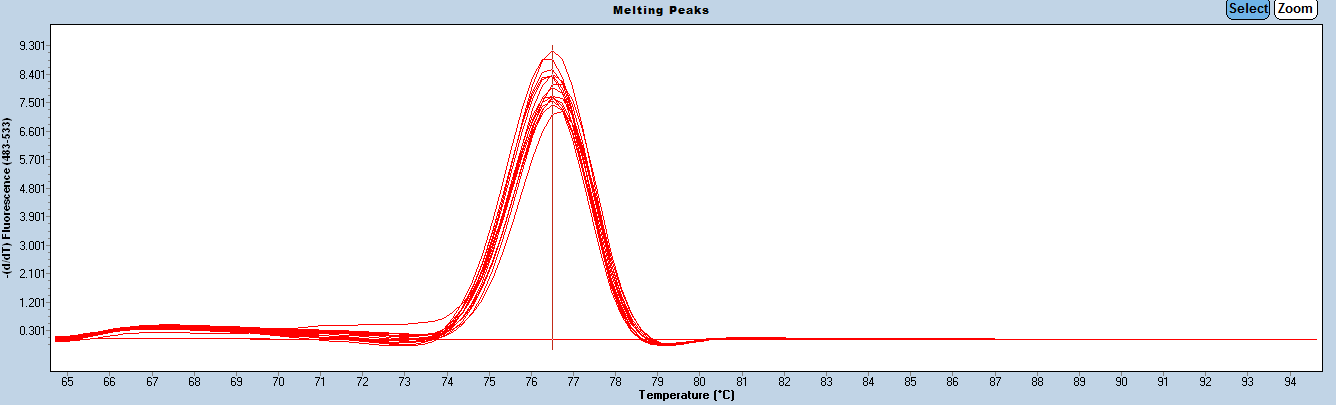


**Figure S1.** Specificity of the circRNA RT-PCR products: Melting curves and agarose gel electrophoresis. **I**) Melting curve analyses of (A) circEGLN3, (B) circNOX4, and (C) circRHOBTB3 specific RT-qPCR products on the LightCycler 480. (**A**) Melting curve of circEGLN3. A single melting peak at 82.5°C confirms the reliability of primer design and PCR run conditions for quantification of circEGLN3. An additional data acquisition step was included in the cycling protocol to cut the fluorescence readings caused by the generation of primer–dimers as shown in the curve. (**B**) Melting curve of circNOX4. A single melting peak at 79.0°C confirms the reliability of primer design and PCR run conditions for quantification of circNOX4. (**C**) Melting curve circRHOBTB3. A single melting peak at 76.5°C confirms the reliability of primer design and PCR run conditions for quantification of circRHOBTB3.


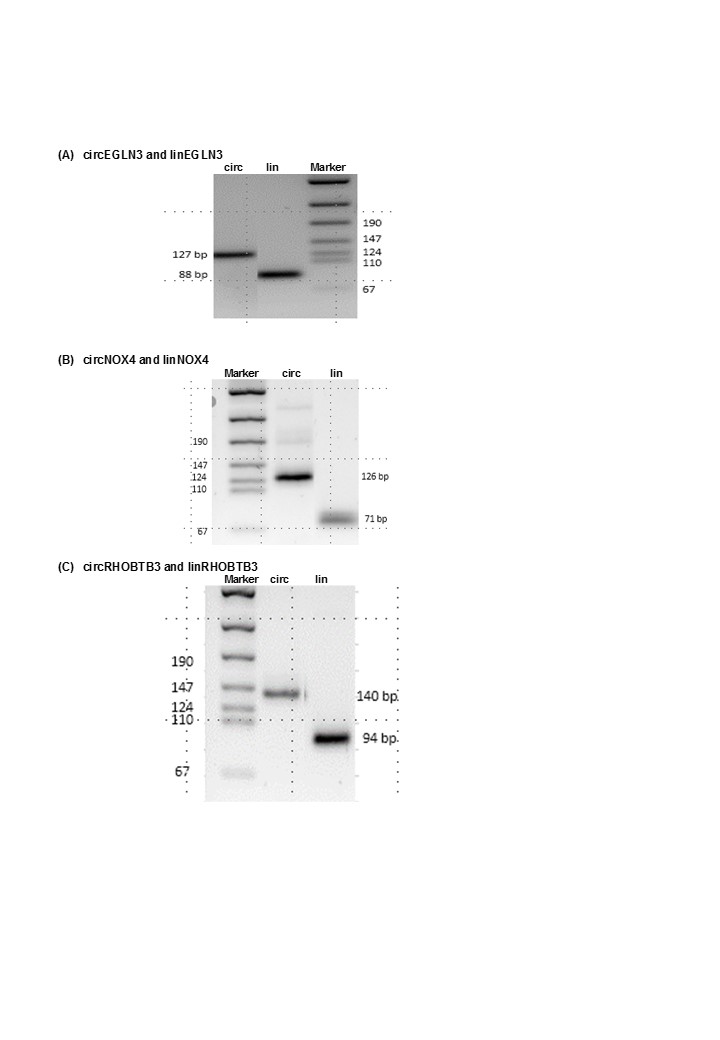


**Figure S1.** Specificity of the circRNA RT-PCR products: Melting curves and agarose gel electrophoresis. II) Agarose gel electrophoresis of RT-qPCR products**.** Gel electrophoresis was performed with 3% agarose gel (Biozyme Small DNA low melt Agarose) on a Biometra Agarose Mini chamber. Analytical details: TBE-running and gel buffer both including ethidium bromide (0.5 µg/ml); gel size: 7x10 cm; 0.5 cm thick; per lane: 8 µl amplicon (or diluted) + 2 µl Gel Loading Solution (Sigma, Traufkirchen, Germany, Cat. No. G2526); run conditions: 100 min, 88 V. Images were documented with Multi Imager System Fusion FX7 (Vilber Lourmat, Eberhardzell, Germany). Fragment sizes were calculated with Fusion Capt Advance Software using the eight shortest markers of the DNA Molecular Weight Marker VIII set (Roche Diagnostics, Cat.No. 11336045001).

**Table S3.** Characteristics of the standard curves of qPCR analyses. Standard curves were generated either from diluted cDNAs or from diluted amplicons. Cq values were calculated by the LightCycler Software Version 1.5.0 using the "second derivative maximum" method. The efficiency, the slope, intercept, and error of the regression line as well as the so-called dynamic range were calculated by the LightCycler480 software.

| **Gene** | **PCR efficiency ^a^** | **Slope** | **y-Intercept** | **Error ^b^** | **Dynamic range ^c^** | **Cq range of the measured samples ^d^** |
| --- | --- | --- | --- | --- | --- | --- |
|  |  |  |  |  |  |  |
| **circEGLN3** | 1.980 | -3.367 | 24.27 | 0.0295 | 24.10 - 33.70 | 21.94 - 33.93 |
| **circNOX4** | 2.015 | -3.286 | 17.27 | 0.0218 | 17.52 - 34.71 | 20.14 - 29.90 |
| **circRHOBTB3** | 2.190 | -2.938 | 20.75 | 0.0466 | 20.96 - 32.76 | 22.51 - 28.91 |
| **linEGLN3** | 1.922 | -3.523 | 14.39 | 0.0112 | 14.40 - 34.90 | 16.86 - 28.45 |
| **linNOX4** | 1.936 | -3.487 | 19.49 | 0.0243 | 19.44 - 36.31 | 23.70 - 32.09 |
| **linRHOBTB3** | 1.880 | -3.648 | 21.96 | 0.0354 | 21.96 - 33.40 | 21.60 - 28.10 |
| **PPIA** | 1.931 | -3.499 | 17.70 | 0.00403 | 17.80 - 32.30 | 18.04 - 22.18 |
| **TBP** | 1.840 | -3.777 | 22.51 | 0.000786 | 22.20 - 32.20 | 23.74 - 28.40 |

^a^ The PCR-efficiency is calculated by the LightCycler480 software after the formula: Efficiency=10^-1/slope^. ^b^ The error value is the mean squared error of the single data points fit to the regression line according to the LightCycler 480 operator’s manual. ^c^ Dynamic range represents the range of mean Cq values between the highest and the lowest Cq values of the generated standard curve. ^d^ Cq range of the measured samples represents the lowest and highest Cq values that were measured in samples of this study.

Supplementary Information S3: circRNA validation methods

Specific Primer Design for circRNAs

CircRNAs are covalently closed RNA circles, therefor divergent primers are necessary for specific detection in RT-qPCR [4]. As primer template serves the individual circRNA backsplice junction. Backsplice junction sequences are unique for every circRNA and ensure specific detection of the circular product in RT-qPCR. All primers were designed using the blasting tool provided by Primer3 [5] and were produced by TIB MOLBIOL (Berlin, Germany). The backsplice junction sequences and divergent primer pairs for all three circRNAs are listed in the following Supplemental Table S4.

**Table S4.** List of backsplice junctions and primers used for RT-qPCR detection of circEGLN3, circNOX4, and circRHOBTB3.

|  | **Backsplice junction sequence** | **Forward primer** | **Reverse primer** |
| --- | --- | --- | --- |
| circEGLN3 | **TCCTGCAGACATCCTACTCG**GCCAGCGGTTTACCTGATAGATTCGGCAATGGTGGCTTGCTATCCGGGAAATGGAACAGGTTATGTTCGCCACGTGGACAACCCCAAC**GGTGATGGTCGCTGCATC** | TCCTGCAGACATCCTACTCG | GATGCAGCGACCATCACC |
| circNOX4 | **CATTGTCCCAGTGTATCTGCAT**TAGAAAATCATCCATTTACCCTCACAATGAGGGCTGCTGAAGTATCAAACTAATTTAGATACCCACCCTCCCGGCTGCATCAGT**CTTAACCGAACCAGCTCTCA** | CATTGTCCCAGTGTATCTGCAT | TGAGAGCTGGTTCGGTTAAGA |
| circRHOBTB3 | **TTCTGGGGATGTTTCAAATG**TAATCGAGAAAGTTAAATGCATTTTAAAAACACCAGGAAAGAAAAAATGCCTGTCTTAAAGGCTGAAGCGTCACATTATAACTCTGACTTAAATAACTTG**CTGTTCTGCTGCCAGTGTGT** | TTCTGGGGATGTTTCAAATG | ACACACTGGCAGCAGAACAG |

RNAse R Treatment of Isolated Total RNA

CircRNAs were described to be resistant to the exonuclease RNAse R [3]. RNAse R digests linear RNAs and is used in general to enrich circRNAs before sequencing or RT-qPCR [6,7]. We performed RNAse R treatment with total RNA extracted from malignant and adjacent normal ccRCC tissue samples. We added 3U of RNAse R (Epicentre, Illumina, San Diego, USA) and 10U of Ribonuclease Inhibitor (abm, Richmond, Canada) to 1 µg of total RNA, reaction buffer and water in a total volume of 10 µl [6]. Afterwards, the mixture was incubated at 37°C for 15 minutes. After incubation and addition of Qiazol reagent, C. elegans (8x10^9^ copies) was spiked-in for normalization. Subsequently, RNA isolation steps were performed with RNeasy Mini Kit (Qiagen) as described in Supplementary Information S2. Mock samples were treated with water as control. Next, cDNA was reversely transcribed and RT-qPCR performed (see "cDNA synthesis" described in Information S2). 2^-ΔΔCT^ method was used to calculate fold change between expressions of RNAse R treated and mock treated samples. The samples were normalized to C. elegans spike-in and circRNAs (circ4 and circ9) previously described in literature were determined as control for circRNA enrichment [3,8]. As linear controls mRNA from EGLN3, NOX4, RHOBTB3, GAPDH, and VCL gene loci were determined.

Random Hexamer Primers vs. oligo(dT) Primers for cDNA Synthesis

As covalently closed structures circRNAs lack a poly-A-tail [9]. Thus, random hexamer primers are used for amplification during cDNA synthesis. The total RNA was extracted from ccRCC malignant and adjacent normal tissue samples and cDNA synthesis was performed with Roche Transcriptor First Strand Synthesis Kit (Roche, Mannheim, Germany) using random hexamer primers (Roche, Mannheim, Germany) in comparison to oligo(dT) primers (Roche, Mannheim, Germany) for circRNA cDNA synthesis. The binding capacity of oligo(dT) primers is reduced without polyadenylated binding sites and reduced quantitation cycle (ΔCq) values result in RT-qPCR when using random hexamer primers in comparison to oligo(dT) primers for circRNA cDNA synthesis. Subsequently, RT-qPCR measurements of circRNAs (circEGLN3, circNOX4, and circRHOBTB3) and mRNAs as controls (GAPDH, TMEM45A) were performed.

Gel Electrophoresis of Specific qPCR Products Obtained with Divergent and Convergent Primers

CircRNAs are products of co- or posttranscriptional backsplicing processes [3,4]. Backspliced circRNA sequences can be reversely transcribed to complementary DNA (cDNA) which allows sequence detection and amplification with divergent primers in RT-qPCR. Consequently, circRNA detection via divergent primer alignment cannot be performed in genomic DNA (gDNA). We performed RT-qPCR with divergent primers for circRNA detection in cDNA and gDNA. As a control, we designed convergent primers that allow sequence amplification of circRNA composing exons in cDNA and gDNA. Convergent primers were designed using software Primer3 [5]. Exon sequences that are part of the respective circRNA were used as templates for designing specific primers. Product length was controlled with agarose gel electrophoresis (Figure 3). All products resembled the expected product length and no by-products were detected in electrophoresis.

Sanger-Sequencing of circRNA Backsplice Junction

Connection of a downstream 3’ end and an upstream 5’ end creates a unique backsplice junction sequence for every circRNA [3,4]. By sequencing the backsplice junction circRNAs can be identified and verified. For sequencing, we first performed RT-qPCR amplification with divergent primers and subsequently determined lengths of the resulting products by gel electrophoresis. Then, products were purified and Sanger sequencing was performed by LGC (Berlin, Germany). For sequencing, same primers were used as for RT-qPCR. Since forward primers for circEGLN3 and circNOX align to a sequence less than 30 bp upstream from the backsplice junction results are prone to error. Sanger Sequencing was performed with revers primers to control results. Backsplice junction sequences (Supplemental Table S4) were validated by Sanger sequencing for all three circRNAs and are shown in Figure 3.

Supplementary Information S4: Associations between clinicopathological variables and circRNAs/linear transcripts

**Table S5.** Associations of circRNAs and linear transcripts with clinicopathological variables. The associations were calculated by Mann-Whitney U-test, Kruskal- Wallis test or Spearman rank correlations coefficient and indicated by *p*-values.

| **Variables** | **Test** | **circEGLN3** | **circNOX4** | **circRHOBTB3** | **linEGLN3** | **linNOX4** | **linRHOBTB3** |
| --- | --- | --- | --- | --- | --- | --- | --- |
| **TNM stage** | KW | *p* = 0.605 | *p* = 0.320 | *p* = 0.305 | *p* = 0.952 | *p* = 0.711 | *p* = 0.051 |
| **TNM stage grouping** | KW | *p* = 0.413 | *p* = 0.282 | *p* = 0.769 | *p* = 0.796 | *p* = 0.603 | *p* = 0.198 |
| **Fuhrman grade** | KW | *p* = 0.006 | *p* = 0.566 | *p* = 0.992 | *p* = 0.004 | *p* = 0.347 | *p* = 0.244 |
| **Surgical margin** | MW | *p* = 0.343 | *p* = 0.995 | *p* = 0.868 | *p* = 0.352 | *p* = 0.778 | *p* = 0.089 |
| **Metastasis** | MW | *p* = 0.270 | *p* = 0.978 | *p* = 0.711 | *p* = 0.550 | *p* = 0.882 | *p* = 0.109 |
| **Tumor size** | SP | *p* = 0.305 | *p* = 0.231 | *p* = 0.539 | *p* = 0.284 | *p* = 0.403 | *p* = 0.110 |
| **Age** | SP | *p* = 0.093 | *p* = 0.887 | *p* = 0.208 | *p* = 0.182 | *p* = 0.551 | *p* = 0.371 |
| **Sex** | MW | *p* = 0.653 | *p* = 0.113 | *p* = 0.852 | *p* = 0.446 | *p* = 0.268 | *p* = 0.852 |

Abbreviations: KW = Kruskal-Wallis test with the Jonckheere-Terpstra trend test; MW = Mann-Whitney U-test; SP = Spearman rank correlation coefficient.

**Table S6a.** Spearman rank correlation coefficients between circRNAs and linear transcripts in adjacent normal ccRCC samples (*n* = 85).

| **RNA** | **r_s_** | **circEGLN3** | **circNOX4** | **circRHOBTB3** | **linEGLN3** | **linNOX4** | **linRHOBTB3** |
| --- | --- | --- | --- | --- | --- | --- | --- |
| **circEGLN3** | r_s_  *p*-value |  | −0.102 0.354 | 0.500 <0.0001 | 0.624 <0.0001 | −0.245 0.024 | 0.488 <0.0001 |
| **circNOX4** | r_s_  *p*-value | −0.102 0.354 |  | 0.051 0.642 | −0.145 0.185 | 0.851 <0.0001 | −0.222 0.042 |
| **circRHOBTB3** | r_s_  *p*-value | 0.500 <0.0001 | 0.051 0.642 |  | 0.286 0.008 | -0.184 0.093 | 0.749 <0.0001 |
| **linEGLN3** | r_s_  *p*-value | 0.624 <0.0001 | −0.145 0.185 | 0.286 0.008 |  | −0.228 0.036 | 0.396 0.0002 |
| **linNOX4** | r_s_  *p*-value | −0.245 0.024 | 0.851 <0.0001 | −0.184 0.093 | −0.228 0.036 |  | −0.301 0.005 |
| **linRHOBTB3** | r_s_  *p*-value | 0.488 <0.0001 | −0.222 0.042 | 0.749 <0.0001 | 0.396 0.0002 | −0.301 0.005 |  |

**Table S6b.** Spearman rank correlation coefficients between circRNAs and linear transcripts in malignant samples (*n* = 99).

| **RNA** | **r_s_** | **circEGLN3** | **circNOX4** | **circRHOBTB3** | **linEGLN3** | **linNOX4** | **linRHOBTB3** |
| --- | --- | --- | --- | --- | --- | --- | --- |
| **circEGLN3** | r_s_  *p*-value |  | 0.289 0.004 | −0.024 0.817 | 0.742 <0.0001 | 0.134 0.187 | −0.261 0.009 |
| **circNOX4** | r_s_  *p*-value | 0.289 0.004 |  | 0.097 0.339 | 0.265 0.008 | 0.849 <0.0001 | −0.047 0.641 |
| **circRHOBTB3** | r_s_  *p*-value | −0.024 0.817 | 0.097 0.339 |  | −0.022 0.829 | 0.085 0.401 | 0.848 <0.0001 |
| **linEGLN3** | r_s_  *p*-value | 0.742 <0.0001 | 0.265 0.008 | −0.022 0.829 |  | 0.158 0.119 | −0.252 0.012 |
| **linNOX4** | r_s_  *p*-value | 0.134 0.187 | 0.849 <0.0001 | 0.085 0.401 | 0.158 0.119 |  | 0.055 0.586 |
| **linRHOBTB3** | r_s_  *p*-value | −0.261 0.009 | −0.047 0.641 | 0.848 <0.0001 | −0.252 0.012 | 0.055 0.586 |  |

**Table S6c.** Significantly different correlation coefficients of circRNAs and linear transcripts between the malignant and adjacent normal samples. Significantly different correlation coefficients (*p* <0.05) between the two specimens were indicated by *p*-values. The yellow-highlighted correlations were not only differently but also inversely correlated (see Supplemental Tables S6A und S6B).

|  | **circEGLN3** | **circNOX4** | **circRHOBTB3** | **linEGLN3** | **linNOX4** | **linRHOBTB3** |
| --- | --- | --- | --- | --- | --- | --- |
| **circEGLN3** | - | 0.008 | <0.0001 | - | 0.011 | <0.0001 |
| **circNOX4** | 0.008 | - | - | 0.006 | - | - |
| **circRHOBTB3** | <0.0001 | - | - | 0.036 | - | - |
| **linEGLN3** | - | 0.006 | - | - | 0.009 | <0.0001 |
| **linNOX4** | 0.011 | - | - | 0.009 | - | 0.015 |
| **linRHOBTB3** | <0.0001 | - | - | <0.001 | 0.015 | - |

Supplementary Information S5: Univariate and multivariate Cox regression analyses as well as Kaplan-Meier analyses

**Table S7.** Univariate Cox regression analyses of circRNAs and linRNAs with their cutoffs predicting cancer-specific, overall and recurrence-free survival. Optimized cutoffs calculated by X-tile software [10] for the corresponding outcome are indicated in parentheses under the name of the variables and explained in the footnotes a, b, and c.

| **Variables**  **(cutoff)** | **Cancer-Specific Survival** | | **Recurrence-Free Survival** | | **Overall Survival** | |
| --- | --- | --- | --- | --- | --- | --- |
|  | **Hazard ratio (95% CI)** | ***p-*value** | **Hazard ratio (95% CI)** | ***p* value** | **Hazard ratio (95% CI)** | ***p-*value** |
| circEGLN3 ^a^  (2.65) | 0.22  (0.10-0.51) | 0.0004 | – | – | – | – |
| circEGLN3 ^b^  (2.10) | – | – | 0.25  (0.11-0.62) | 0.003 | – | – |
| circEGLN3 ^c^  (2.73) | – | – | – | – | 0.34  (0.17–0.67) | 0.002 |
| circNOX4 ^a^  (0.25) | 0.51  (0.20–1.26) | 0.147 | – | – | – | – |
| circNOX4 ^b^  (0.27) | – | – | 0.57  (0.26–1.25) | 0.163 | – | – |
| circNOX4 ^c^  (0.31) | – | – | – | – | 0.41  (0.16–1.05) | 0.064 |
| circRHOBTB3 ^a^  (0.20) | 0.36  (0.13–0.95) | 0.039 | – | – | – | – |
| circRHOBTB3 ^b^  (0.50) | – | – | 0.58  (0.25–1.34) | 0.205 | – | – |
| circRHOBTB3 ^c^  (0.27) | – | – | – | – | 0.46  (0.23–0.93) | 0.031 |
| linEGLN3 ^a^  (2.80) | 0.34  (0.15–0.78) | 0.011 | – | – | – | – |
| linEGLN3 ^b^  (3.05) | – | – | 0.33  (0.14–0.78) | 0.012 | – | – |
| linEGLN3 ^c^  (3.0) | – | – | – | – | 0.50  (0.25–1.01) | 0.052 |
| linNOX4 ^a^  (0.71) | 0.69  (0.29–1.64) | 0.399 | – | – | – | – |
| linNOX4 ^b^  (1.10) | – | – | 2.09  (0.74–5.45) | 0.170 | – | – |
| linNOX4 ^c^  (0.53) | – | – | – | – | 1.26  (0.68–2.34) | 0.459 |
| linRHOBTB3 ^a^  (1.52) | 2.75  (1.09–6.98) | 0.033 | – | – | – | – |
| linRHOBTB3 ^b^  (0.72) | – | – | 3.57  (1.49–8.55) | 0.043 | – | – |
| linRHOBTB3 ^c^  (0.60) | – | – | – | – | 1.87  (0.99–3.53) | 0.053 |

^a^ Optimized cutoff (given as normalized arbitrary expression units) calculated by using the software X-tile for cancer-specific survival but also applied in the analyses of overall and recurrence-free survival. ^b^ Optimized cutoff (given as normalized arbitrary expression units) calculated by using the software X-tile for recurrence-free survival. ^c^ Optimized cutoff (given as normalized arbitrary expression units) calculated by using the software X-tile for overall survival.


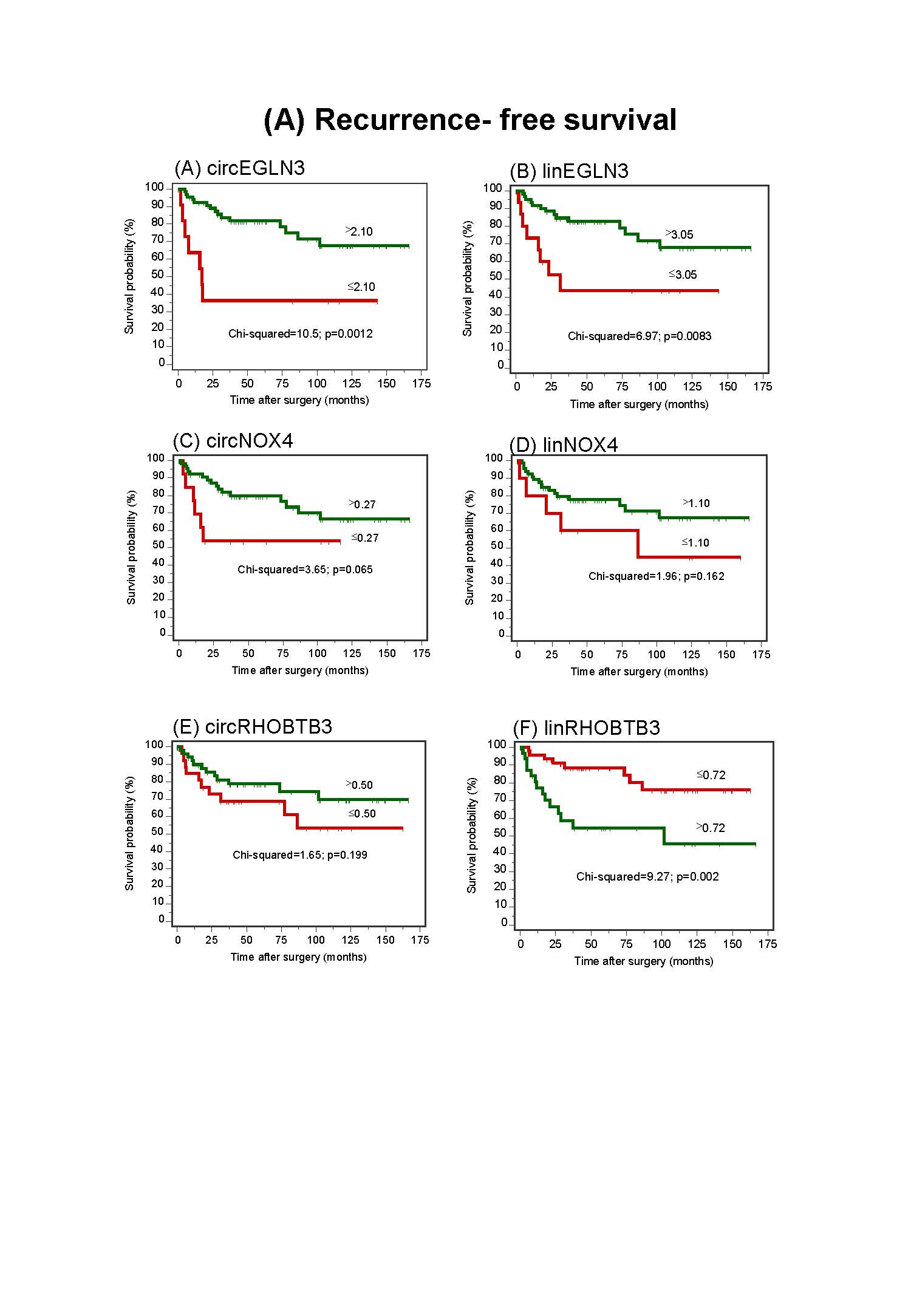


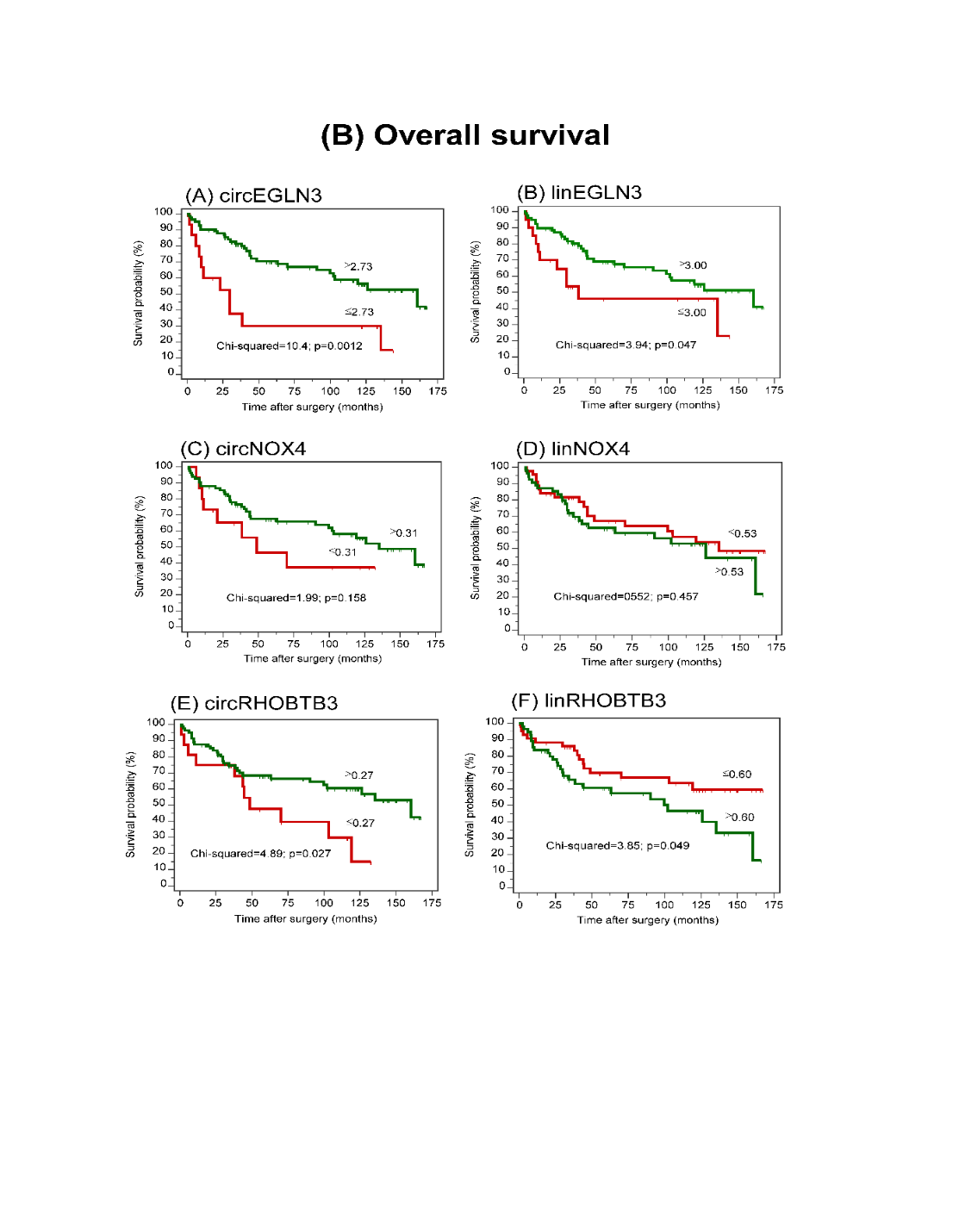


**Figure S2.** Kaplan-Meier curves of circRNAs and linRNAs in association with (A) recurrence-free and (B) overall survival. The categorization of the variables by dichotomization is indicated in the figures.


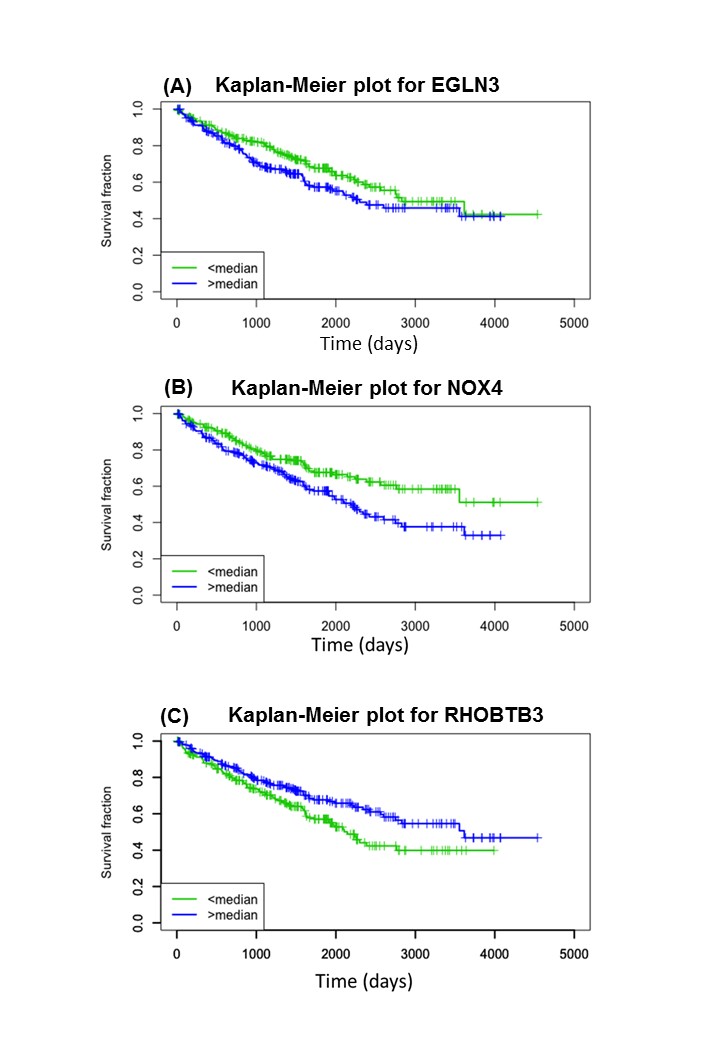


**Figure S3A-C.** Kaplan-Meier analysis of mRNAs of EGLN3, NOX4, and RHOBTB3 with regard to overall survival after surgery of ccRCC patients from the TCGA data set.

**Table S8.** Univariate Cox regression analyses of clinicopathological factors predicting cancer-specific, recurrence-free and overall survival. Either continuous values or binary categories indicated with their cutoffs in parentheses under the name of the variable were used for calculations. Variables highlighted in yellow with insignificant *p*-values were eliminated using only the four remaining variables as "clinical model" variables in further multivariate Cox regression analyses together with circRNAs and linRNAs.

| **Variables** | **Cancer-Specific Survival** | | **Recurrence-Free Survival** | | **Overall Survival** | |
| --- | --- | --- | --- | --- | --- | --- |
|  | **Hazard ratio (95% CI)** | ***p-*value** | **Hazard ratio (95% CI)** | ***p-*value** | **Hazard ratio (95% CI)** | ***p-*value** |
| Age  (yrs, continuous) | 0.99  (0.96–1.02) | 0.573 | 0.99  (0.96–1.03) | 0.846 | 1.01  (0.99–1.04) | 0.326 |
| Sex  (female/male) | 1.28  (0.57–2.88) | 0.551 | 1.08  (0.44–2.65) | 0.869 | 1.03  (0.53–2.03) | 0.921 |
| Tumor size  (≥7cm<) | 3.33  (1.49–7.44) | 0.003 | 2.92  (1.26–6.76) | 0.012 | 2.24  (1.18–4.25) | 0.013 |
| Tumor stage grouping (stage III-IV/stage I-II) | 6.26  (2.33–16.8) | 0.0003 | 2.86  (1.22–6.70) | 0.016 | 3.07  (1.61–5.85) | 0.0006 |
| Fuhrman grading  (G3-4/G1-2) | 6.03  (2.64–13.8) | <0.0001 | 15.3  (5.92–39.9) | <0.0001 | 4.85  (2.38–9.87) | <0.0001 |
| Surgical margin  (R1 vs R0) | 8.02  (3.48–18.5) | <0.0001 | 10.2  (3.84–27.0) | <0.0001 | 5.10  (2.38–10.9) | <0.0001 |
| Primary metastasis (yes/no) | 9.31  (4.08–21.2) | <0.0001 |  |  | 6.01  (2.88–12.5) | <0.0001 |
| Surgery (radical/partial) | 1.16  (0.39–3.39) | 0.790 | 1.59  (0.58–4.35) | 0.362 | 0.72  (0.28–1.86) | 0.492 |


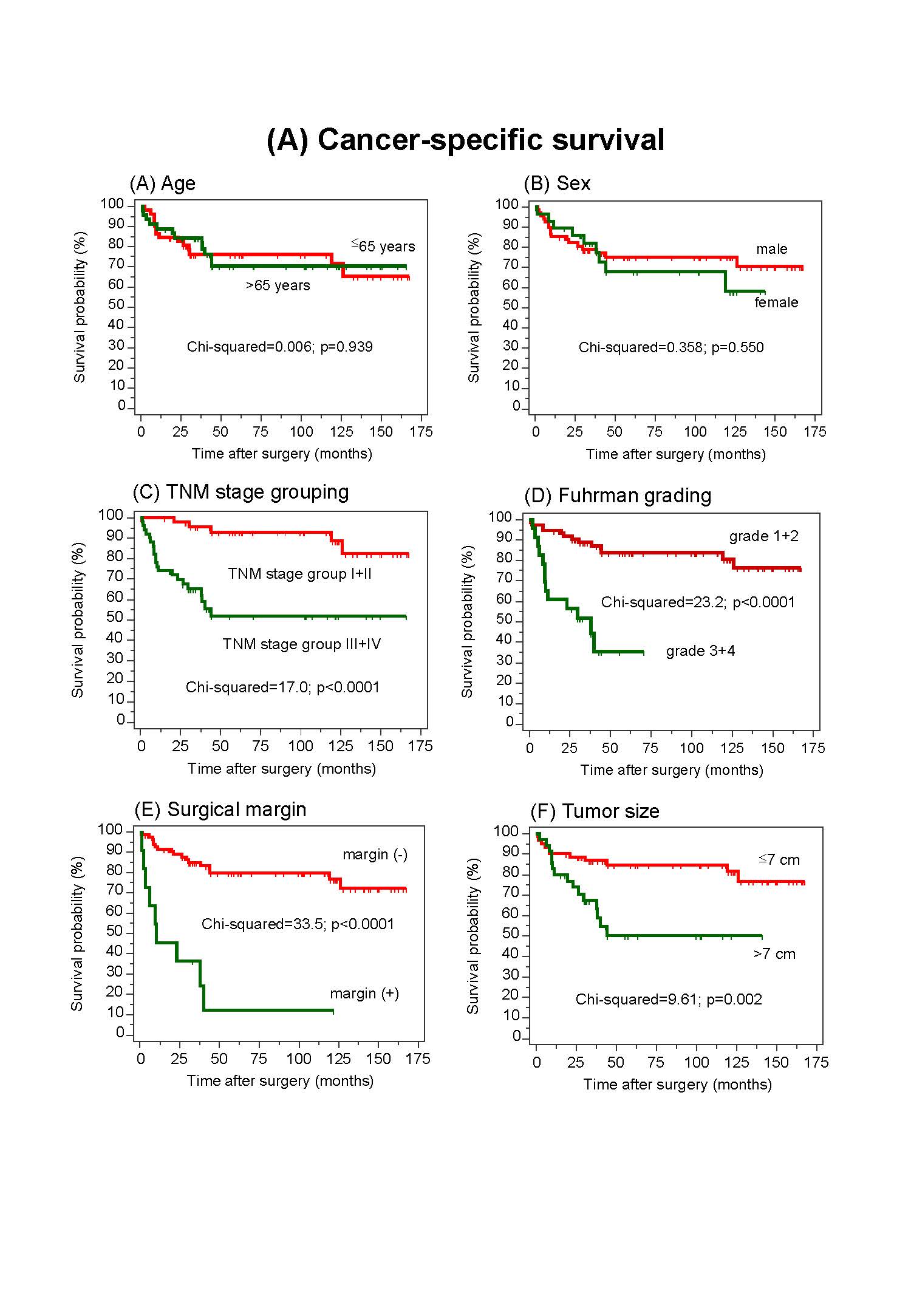


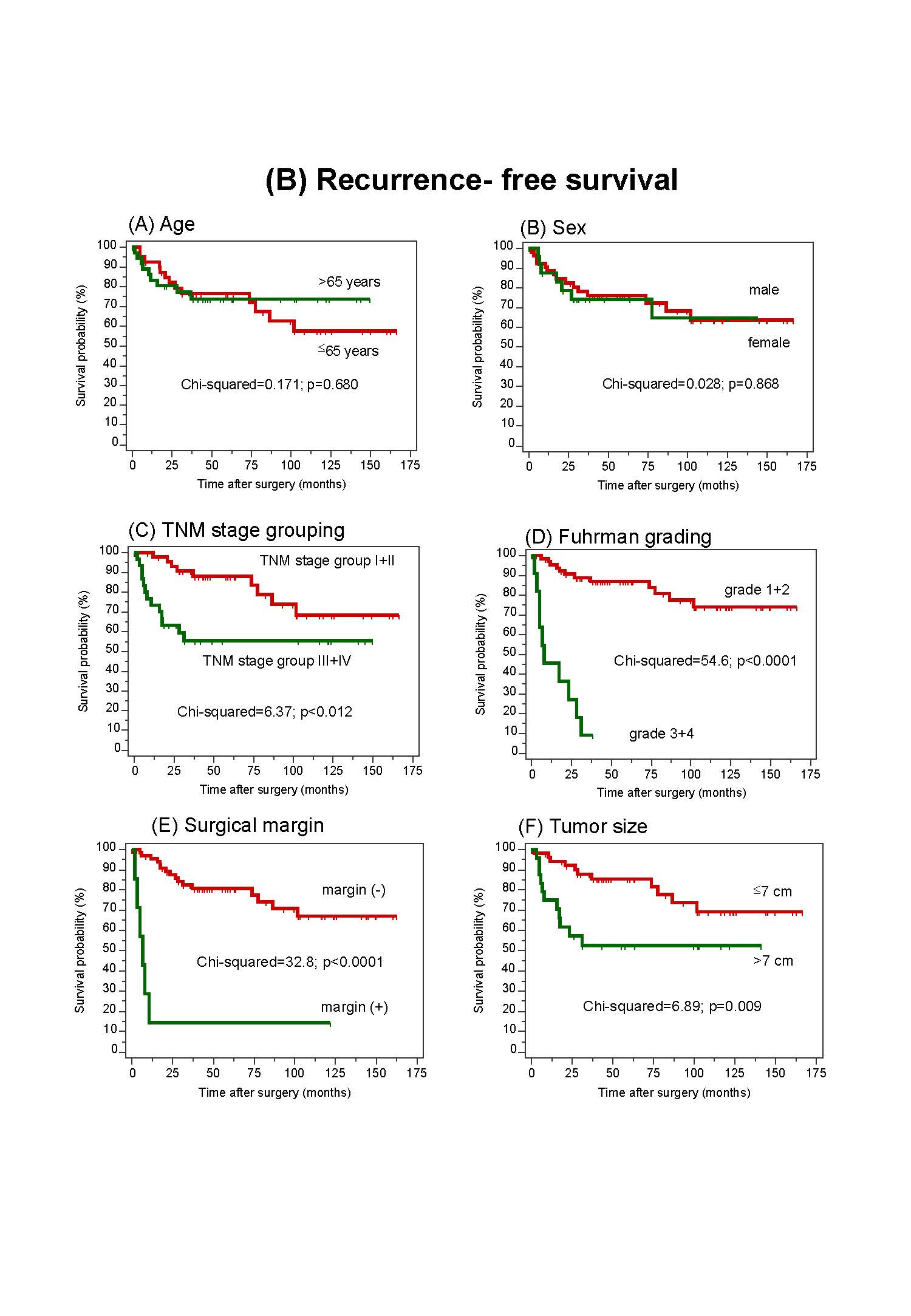


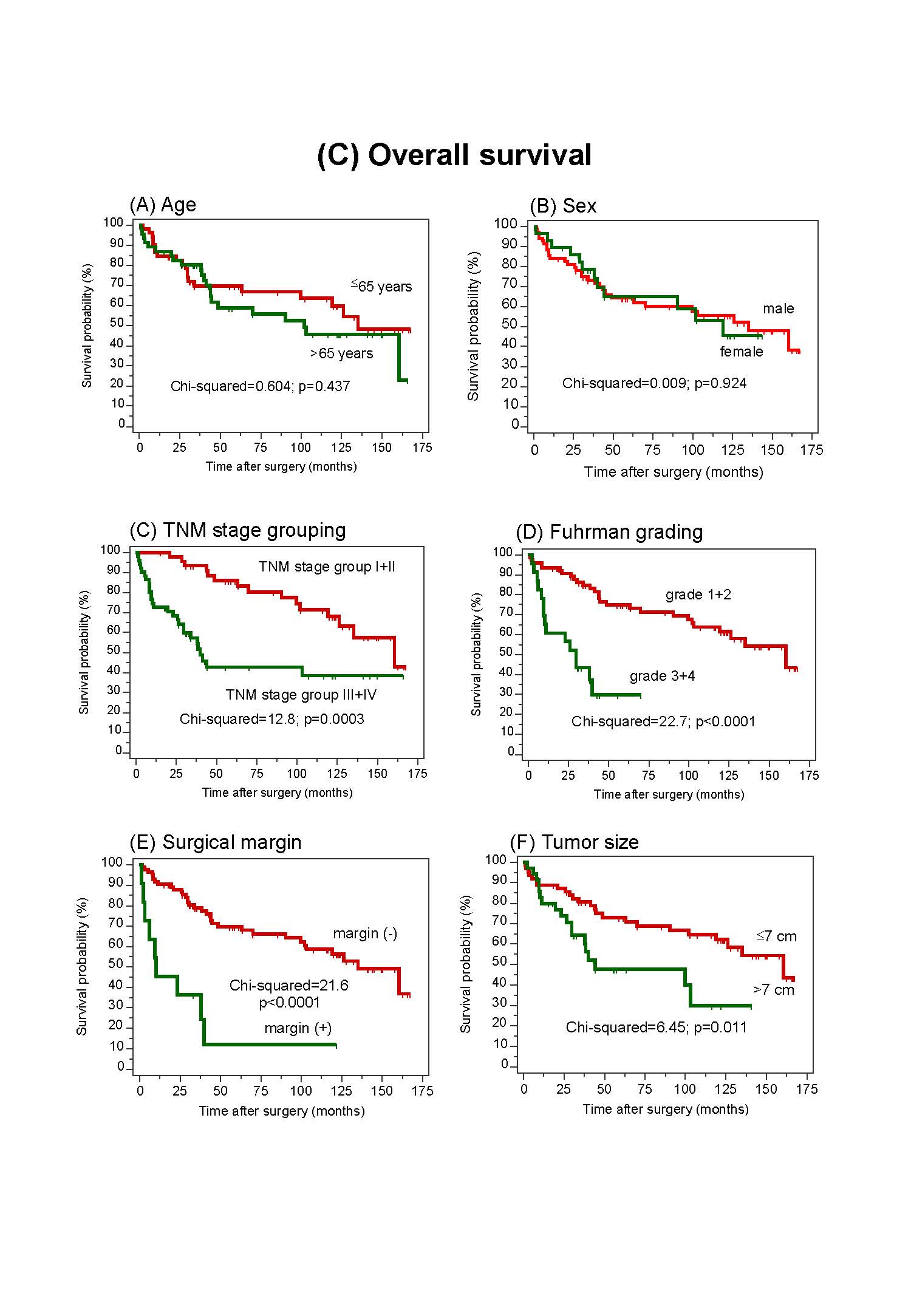


**Figure S4.** Kaplan-Meier curves of clinicopathological factorsin association with (**A**) cancer-specific, (**B**) recurrence-free, and (**C**) overall survival. The categorization of the variables by dichotomization is indicated in the figures.

**Table S9.** Bootstrapping *p* values of multivariate Cox regression analyses using different models for predicting cancer-specific, recurrence-free, and overall survival in ccRCC patients after nephrectomy ^a^.

| **Variable ^b^** | **Cancer-Specific Survival** | **Recurrence-Free Survival** | **Overall Survival** |
| --- | --- | --- | --- |
|  | ***p-*value** | ***p-*value** | ***p-*value** |
| RNA signature ^c,d,e^ | | | |
| circEGLN3 | 0.003 | 0.314 | 0.048 |
| circRHOBTB3 | 0.005 | 0.004 | 0.003 |
| linRHOBTB3 | 0.095 | <0.001 | 0.010 |
| *Clinical model* | | | |
| Tumor stage grouping (III+IV/I+II) | 0.009 | 0.693 | 0.080 |
| Fuhrman grading (G3+4/G1+2) | 0.057 | <0.0001 | 0.015 |
| Surgical margin (R1/R0) | 0.047 | 0.008 | 0.107 |
| Tumor size (≥7cm<) | 0.869 | 0.925 | 0.989 |
| Clinical model + RNA signature | | | |
| Tumor stage grouping (III+IV/I+II) | 0.068 | 0.589 | 0.032 |
| Fuhrman grading (3+4/1+2) | 0.251 | <0.0001 | 0.129 |
| Surgical margin (R1 vs. R0) | 0.018 | 0.230 | 0.159 |
| Tumor size (≥7cm<) | 0.795 | 0.778 | 0.588 |
| circEGLN3 | 0.013 | 0.571 | 0.113 |
| circRHOBTB3 | 0.039 | 0.048 | 0.002 |
| linRHOBTB3 | 0.023 | 0.031 | 0.005 |
| Clinical model + RNA signature after backward elimination | | | |
| Tumor stage grouping (III+IV/I+II) | 0.007 | – | 0.005 |
| Fuhrman grading (3+4/1+2) | – | <0.001 | 0.043 |
| Surgical margin (R1 vs. R0) | <0.001 | 0.098 | – |
| Tumor size (≥7cm<) | – | – | – |
| circEGLN3 | 0.001 | – | 0.036 |
| circRHOBTB3 | 0.009 | 0.045 | 0.003 |
| linRHOBTB3 | 0.019 | 0.037 | 0.004 |

Abbreviations: ccRCC, clear cell renal cell carcinoma; G, histopathological grading according to Fuhrman; R, surgical margin classification. The footnotes a–e correspond to those in Table 5 of the main text.

References

1. Bustin, S.A.; Benes, V.; Garson, J.A.; Hellemans, J.; Huggett, J.; Kubista, M.; Mueller, R.; Nolan, T.; Pfaffl, M.W.; Shipley, G.L.; et al. The MIQE guidelines: minimum information for publication of quantitative real-time PCR experiments. *Clin. Chem.* **2009**, *55*, 611–622.
2. Jung, M.; Ramankulov, A.; Roigas, J.; Johannsen, M.; Ringsdorf, M.; Kristiansen, G.; Jung, K. In search of suitable reference genes for gene expression studies of human renal cell carcinoma by real-time PCR. *BMC Mol. Biol.* **2007,** *8*, 47.
3. Memczak, S.; Jens, M.; Elefsinioti, A.; Torti, F.; Krueger, J.; Rybak, A.; Maier, L.; Mackowiak, S. D.; Gregersen, L.H.; Munschauer, M.; et al. Circular RNAs are a large class of animal RNAs with regulatory potency. *Nature* **2013**, *495*, 333–338.
4. Jeck, W.R.; Sharpless, N.E. Detecting and characterizing circular RNAs. *Nat. Biotechnol.* **2014**, *32*, 453–461.
5. Untergasser, A.; Cutcutache, I.; Koressaar, T.; Ye, J.; Faircloth, B.C.; Remm, M.; Rozen, S.G. Primer3 - new capabilities and interfaces. *Nucleic Acids Res.* **2012**, *40*, e115.
6. Salzman, J.; Chen, R.E.; Olsen, M.N.; Wang, P.L.; Brown, P.O. Cell-type specific features of circular RNA expression. *PLoS Genet.* **2013**, *9*, e1003777.
7. Szabo, L.; Salzman, J. Detecting circular RNAs: bioinformatic and experimental challenges. *Nat. Rev. Genet.* **2016**, *17*, 679–692.
8. Memczak, S.; Papavasileiou, P.; Peters, O.; Rajewsky, N. Identification and characterization of circular RNAs as a new class of putative biomarkers in human blood. *PLoS One* **2015**, *10*, e0141214.
9. Jeck, W.R.; Sorrentino, J.A.; Wang, K.; Slevin, M.K.; Burd, C.E.; Liu, J.; Marzluff, W. F.; Sharpless, N.E. Circular RNAs are abundant, conserved, and associated with ALU repeats. *RNA* **2013,** *19*, 141–157.
10. Camp, R.L.; Dolled-Filhart, M.; Rimm, D.L. X-tile: a new bio-informatics tool for biomarker assessment and outcome-based cut-point optimization. *Clin. Cancer Res.* **2004,** *10*, 7252–7259.
